# Clinical and molecular characterization of a patient with mitochondrial Neurogastrointestinal Encephalomyopathy

**DOI:** 10.1186/s12876-020-01280-5

**Published:** 2020-05-08

**Authors:** Parham Habibzadeh, Mohammad Silawi, Hassan Dastsooz, Shima Bahramjahan, Shahrokh Ezzatzadegan Jahromi, Vahid Reza Ostovan, Majid Yavarian, Mohammad Mofatteh, Mohammad Ali Faghihi

**Affiliations:** 1 Persian BayanGene Research and Training Center, Shiraz University of Medical Sciences, Shiraz, Iran; 2 Student Research Committee, Shiraz University of Medical Sciences, Shiraz, Iran; 3 Italian Institute for Genomic Medicine (IIGM), University of Turin, Turin, Italy; 4 Shiraz Nephro-Urology Research Center, Shiraz University of Medical Sciences, Shiraz, Iran; 5 Clinical Neurology Research Center, Shiraz University of Medical Sciences, Shiraz, Iran; 6 Sir William Dunn School of Pathology, University of Oxford, Oxford, UK; 7 Department of Psychiatry and Behavioral Sciences, University of Miami Miller School of Medicine, Miami, USA

**Keywords:** Mitochondrial diseases, Mitochondrial neurogastrointestinal encephalomyopathy syndrome, *TYMP*, Codon, nonsense

## Abstract

**Background:**

Mitochondrial neurogastrointestinal encephalomyopathy (MNGIE) is a rare autosomal recessive disorder caused by mutations in *TYMP* gene, encoding nuclear thymidine phosphorylase (TP). MNGIE mainly presents with gastrointestinal symptoms and is mostly misdiagnosed in many patients as malabsorption syndrome, inflammatory bowel disease, anorexia nervosa, and intestinal pseudo-obstruction. Up to date, more than 80 pathogenic and likely pathogenic mutations associated with the disease have been reported in patients from a wide range of ethnicities. The objective of this study was to investigate the underlying genetic abnormalities in a 25-year-old woman affected with MNGIE.

**Case presentation:**

The patient was a 25-year-old female referred to our center with the chief complaint of severe abdominal pain and diarrhea for 2 years that had worsened from 2 months prior to admission. The clinical and para-clinical findings were in favor of mitochondrial neurogastrointestinal encephalomyopathy syndrome. Subsequent genetic studies revealed a novel, private, homozygous nonsense mutation in *TYMP* gene (c. 1013 C > A, p.S338X). Sanger sequencing confirmed the new mutation in the proband. Multiple sequence alignment showed high conservation of amino acids of this protein across different species.

**Conclusion:**

The detected new nonsense mutation in the *TYMP* gene would be very important for genetic counseling and subsequent early diagnosis and initiation of proper therapy. This novel pathogenic variant would help us establish future genotype-phenotype correlations and identify different pathways related to this disorder.

## Background

Mitochondrial neurogastroinstestinal encephalomyopathy (MNGIE – OMIM# 603041) is a rare multisystem autosomal recessive disorder caused by homozygous or compound heterozygous mutations in the nuclear-encoded thymidine phosphorylase gene (*TYMP*; 131,222) on chromosome 22q13, the first gene whose role was defined at molecular level in the defects of intergenomic communication [1]. Impairment in this enzyme with resultant decreased enzyme activity leads to accumulation of the enzyme’s substrates, thymidine and deoxyuridine, which in turn leads to an imbalance in the intra-mitochondrial nucleotide pool and multiple deletions, point-specific mutations and depletions in mitochondrial DNA (mtDNA) [2, 3]. Earlier studies have suggested that the accumulation of these nucleosides is the main culprit for the development of the molecular and phenotypic aberrations reported in this disorder [4–6].

First described in 1976, MNGIE usually presents before the age of 30 years with a mean age of onset of 18.5 years. Nevertheless, there have been a few patients where onset was as early as 5 months of age and as late as the fifth decade [7–9]. The disease has a progressive clinical course leading to death at the mean age of 37 [9–12]. The clinical manifestations of MNGIE are severe gastrointestinal dysmotility, cachexia, extraocular muscle weakness with resultant ptosis and ophthalmoplegia, sensorimotor neuropathy, and leukoencephalopathy [13]. More than 120 patients with diagnostic features consistent with MNGIE have so far been reported in the literature, with more than 80 pathogenic and likely pathogenic mutations (https://www.ensembl.org) associated with the disease identified in patients from a wide range of ethnicities [14, 15].

Herein, we report on a patient with MNGIE with a novel homozygous mutation in *TYMP* gene, along with the clinical, laboratory and imaging findings.

## Case presentation

### Clinical presentation

A 25-year-old female was referred to our center with the chief complaint of severe abdominal pain and diarrhea for 2 years that had worsened from 2 months prior to admission. She had significant weight loss during this period; weighing 36.5 kg with a height of 160 cm, her body mass index (BMI) was 14.3 kg/m^2^ at the time of admission. Her past medical and surgical history was only significant for one undocumented episode of seizure at the age of three and appendectomy 3 years before. On interview, she denied fear of weight gain, laxative abuse, and self-induced vomiting. Her parents were consanguineous. There was no history of sibling loss or any similar symptoms in other family members.

Clinical examination revealed a cachectic lady with external ophthalmoplegia, ptosis, right lower quadrant scar of the McBurney (oblique) incision for appendectomy, decreased muscle power in the upper (4/5 MRC muscle scale) and lower (3/5 MRC muscle scale) extremities, and absent deep tendon reflexes. Abdominopelvic sonography revealed mild free fluid in the abdominal cavity and increased thickness in the bowel wall (4.8 mm) and a 16 × 11-mm cortical cyst in the upper pole of the left kidney with thin septation. A diagnostic esophago-gastro-duodenoscopy showed diffuse severe erythema and congestion in the body, fundus, and antrum of the stomach with moderate chronic gastritis in pathologic examination and deformity of the duodenal bulb with decreased folds in D2 part of the duodenum. On colonoscopy, the ileocecal valve was stenotic; biopsy revealed submucosal fibrosis with lymphoid proliferation and focal ulceration. Echocardiography was normal except for mild pericardial effusion.

Her complete blood count indicated the presence of microcytic hypochromic anemia. Biochemistry revealed low total protein level (4.1 g/dl; reference range: 6.6–8.8 g/dl) and albumin (2.2 g/dl; reference range: 3.5–5.2 g/dl). Serum lactate level (35.3 mg/dl; reference range: 4.5–19.8 mg/dl) was elevated. Fecal occult blood test was positive with moderately elevated fecal calprotectin level (58.5 μg/g; reference range: < 15 μg/g) suggestive for the inflammatory process. Cerebrospinal fluid analysis revealed marked elevation of protein level (122 mg/dl; reference range: 15–45 mg/dl). Serology tests for HIV, HBV, and HCV were negative. Serum anti-tissue trans-glutaminase antibodies, anti-phospholipid antibodies, anti-cardiolipin antibodies, lupus anti-coagulants, β_2_ microglobulin, anti-nuclear antibody (ANA), anti-double stranded DNA (anti-dsDNA), C-ANCA, and P-ANCA were also within the normal limits. Serum levels of complement factors were also found to be altered: C_3_ level (65 mg/dl; reference range: 90–180 mg/dl) was decreased; C_4_ level (11.4 mg/dl; reference range: 10–40 mg/dl) was within the lower limit of the normal range. Blood TP activity was not measured for lack of laboratory resources.

**Fig. 1:**
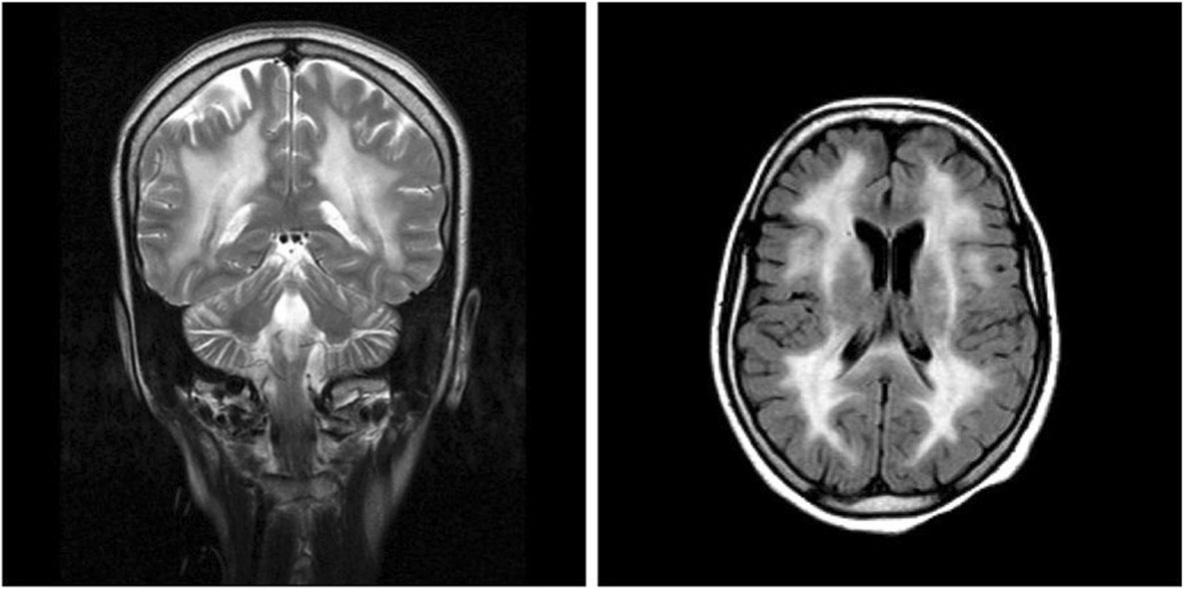
Brain MRI revealing diffuse white matter signal changes

The electrodiagnostic evaluation showed a neurogenic pattern on needle electromyography (EMG), conduction block in sensory nerves, and decreased compound muscle action potential (CMAP) in motor nerves with decreased conduction velocity and prolonged F-wave latency. Brain MRI with contrast showed leukoencephalopathy with diffusely increased T_2_ signal intensity in both cerebral hemispheres white matter. Hypersignal intensity in splenium of corpus callosum was also observed (Fig. 1).

### Molecular analysis

After obtaining informed consent, a blood sample was obtained from the patient. The whole blood sample was collected in EDTA tube. Genomic DNA was then extracted from peripheral leukocytes using the QIAamp DNA Blood Mini Kit (Qiagen, Germany).

Whole Exome Sequencing (WES) was performed on Illumina NextSeq500 instrument to a sequence close to 100 million reads. WES result was then analyzed using different bioinformatics tools and databases such as BWA aligner, GATK and ANNOVAR [16–18]. WES uncovered a novel, private, homozygous stopgain mutation in *TYMP* gene (NM_001113756: exon7: c. 1013C > A: p.S338X, Chr: 50526392).

**Fig. 2:**
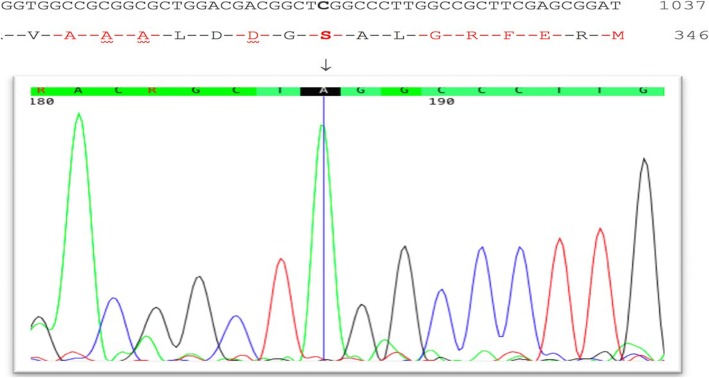
Sanger sequencing electropherogram of the novel identified variant in this study. The arrow shows the position of homozygous mutation in *TYMP* (c. 1013 C > A, p.S338X)

Subsequently in order to confirm this novel mutation using Sanger sequencing, the region of interest was amplified using PCR on the DNA of the proband using following primers: F–TYMP-E7: 5′-ACTTAAGGGACCTGGTCACCAC-3′ and R–TYMP-E7: 5′-AGCCTCTGACCCACGTCGA-3′ (PCR product: 594 bp). Then, the amplicon was sequenced with both forward and reverse primers using ABI BigDye Terminator Cycle Sequencing Kit (Applied Biosystems®, USA). Sanger sequencing result was analyzed by NCBI BLAST (https://blast.ncbi.nlm.nih.gov) and CodonCode Aligner (http://www.codoncode.com/aligner/). Sanger sequencing confirmed this mutation as homozygous in the proband (Fig. 2).

This mutation has not been reported yet in main variant databases and our public database (Bayangene). The mutation occurred in domains described as Glycos_transf_3 in Pfam database or Nucleoside phosphorylase/phosphoribosyltransferase catalytic domain in Gene3D database which affects this domain and other domains such as PYNP_C, Pyrimidine nucleoside phosphorylase C-terminal domain, TIGR02644, THYMIDINE PHOSPHORYLASE and TP_PyNP which are described by a different database.

**Fig. 3:**
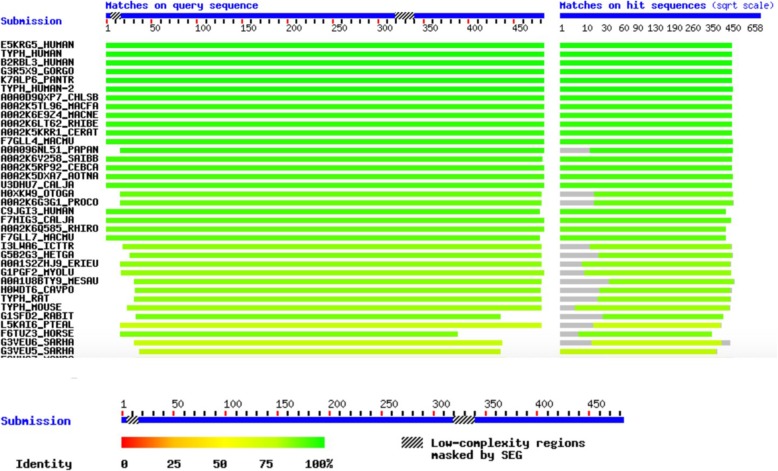
Multiple sequence alignment of the whole amino acid sequence of TYMP with other TYMP orthologs across different species

To reveal the conservation of amino acid sequence of TYMP protein across various species, multiple sequence alignment analysis by BLAST available on ExPASy (https://web.expasy.org/cgi-bin/blast/blast.pl) was also performed. Multiple sequence alignment showed high conservation of amino acids of this protein across different species, mainly in the mutated region and after this position. (Fig. 3).

## Discussion and conclusion

A group of mitochondrial disorders are characterized by mutations in the nuclear genome affecting expression and replication of the genes on the mitochondrial genetic material. Progressive external ophthalmoplegia (PEO) was the first disease identified in this group caused by defects in the intergenomic communication [19]. MNGIE, a rare progressive multisystem autosomal recessive disorder caused by a mutation in *TYMP* gene is also a member of this group of disorders.

**Fig. 4:**
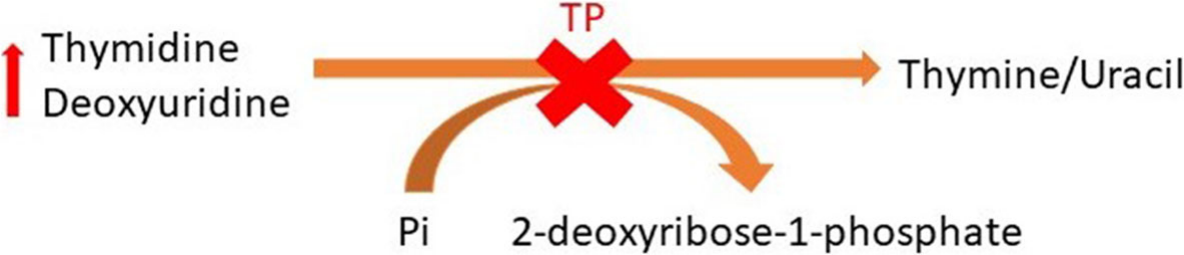
The defect in the chemical reaction catalyzed by the enzyme thymidine phosphorylase in MNGIE

*TYMP* gene which encodes the cytosolic enzyme named thymidine phosphorylase, TP, is located at chromosome 22q13.33. This protein catalyzes phosphorolysis of thymidine and deoxyuridine to 2-deoxyribose 1-phosphate and their corresponding bases (Fig. 4). TP plays both a direct and an indirect role in the metabolic pathways of various cells including those in the brain, muscle, WBCs, and bone marrow [20]. Absence of TP in cells which normally have a high expression of the enzyme (e.g., white blood cells) leads to systemic accumulation of dThd and dUrd which has a toxic effect on other tissues [21]. MNGIE usually presents with symptoms of gastrointestinal dysfunction, such as gastrointestinal motility disorders, gastro-esophageal reflux, dysphagia, abdominal pain and distention, and diarrhea leading to severe weight loss and cachexia [22]. At this stage of the disease, most of the patients are misdiagnosed as malabsorption syndrome, inflammatory bowel disease (IBD), anorexia nervosa, or intestinal pseudo-obstruction, often leading to unnecessary medical interventions and delay in diagnosis of up to 10 years [11, 23–25]. Gastrointestinal signs and symptoms have also been observed in other genetic mitochondrial disorders as well [26]. Ptosis, ophthalmoparesis, hearing loss, and sensory-motor neuropathy constitute the most common neurologic features of patients with MNGIE [13]. Due to the high metabolic activity of extraocular muscles, deterioration in their function resulting in ophthalmoplegia or ophthalmoparesis occurs early in the course of the disease that parallels the disease progression [27]. Neuroimaging studies such as brain MRI and magnetic resonance spectroscopy (MRS) might yield a clue about the diagnosis of MNGIE, with the absence of leukoencephalopathy ruling out MNGIE in most cases. Unlike the patient reported here, who had involvement of splenium of the corpus callosum, it is relatively spared in most individuals [13, 15, 28]. Our patient was found to have leukoencephalopathy, with diffuse T_2_ hyperintensity in both cerebral hemispheres white matter on brain MRI, though, she had normal cognitive function. This, in turn, is likely to be due to the impaired blood-brain barrier function in these patients leading to edema in lieu of demyelination [29].

Other disorders with phenotypes similar to MNGIE, caused by mutations in *RRM2B* and *POLG* genes have been reported [30, 31]. Therefore, it is prudent to test the individuals suspected of having MNGIE for these genes as well. In our patient whole exome sequencing was done and no mutations in these genes were detected.

To the best of our knowledge, around 80 pathogenic mutations in the *TYMP* gene have so far been reported. Attempts to draw a genotype-phenotype correlation in this disorder have mostly been discouraging, except for c. 622G > A variant (p.Val208Met), producing less severe TP dysfunction, leading to a late-onset disease [8, 32, 33].

Current treatment modalities for MNGIE mainly focus on restoration of the activity of TP and lowering the circulatory levels of the nucleoside substrates. Hematopoietic stem cell transplantation (HSCT) has so far been used to restore TP enzyme activity in patients with MNGIE. A retrospective analysis of 24 patients who underwent HSCT for the treatment of MNGIE revealed a survival rate of 37.5% after a median follow-up of almost 4 years. Of the fifteen patients who had died, nine had died from transplant-related mortality, and six from their disease [34]. It was found that younger patients without gastrointestinal dysmotility and liver disease receiving HSCT from an HLA-matched donor would benefit mostly from this type of treatment, highlighting the importance of diagnosis in the momentous days early in the course of the illness, when HSCT would change the course of the disease [34]. Hemodialysis and peritoneal dialysis have also been proposed as treatment modalities in these patients intending to remove the nucleosides from the peripheral circulation. In a 20-year-old patient with MNGIE, peritoneal dialysis was shown to remove approximately 100 μ moles per day of thymidine and 2′-deoxyuridine from the peritoneal cavity. Although there were improvements in the symptoms, nucleosides serum levels remained unchanged [35]. A prospective study evaluating a 29-year-old patient with MNGIE who underwent extensive hemodialysis for 1 year also revealed that it has only a transient effect on the serum and urine levels of nucleosides with no long-term effects; there were no changes in the level of the toxic metabolites in the CSF in both short-term (within 24 h) and long-term (at months 6 and 12) [36]. Our patient in this report underwent hemodialysis with only mild improvements in the gastrointestinal symptoms. These findings cast doubt on the efficacy of dialysis in the treatment of MNGIE. Other therapeutic modalities including platelet infusion, which was also performed in our patient, and orthotopic liver transplantation have also been reported for the treatment of this disease in the literature [37, 38]. Erythrocyte encapsulated thymidine phosphorylase (EE-TP) enzyme replacement therapy which has clinical trial approval is another promising treatment option [39].

The mutation found initially by WES and subsequently confirmed using Sanger sequencing is predicted to disrupt the proper function of TYMP protein since different reports have identified frameshift mutations before and after this region resulting in the impaired TYMP [7, 40, 41].

Our patient had many of the clinical, laboratory, and imaging features seen in MNGIE. The detected novel nonsense mutation in the *TYMP* gene would be of importance for genetic counseling and subsequent early diagnosis and initiation of proper therapy. On account of the wide clinical spectrum of signs and symptoms presented by patients with MNGIE, molecular diagnostic methods would be of paramount importance.

## Data Availability

All data are available from the corresponding author on request.

## Abbreviations

MNGIE: Mitochondrial neurogastrointestinal encephalomyopathy
TP: Thymidine phosphorylase
mtDNA: Mitochondrial DNA
ANA: Anti-nuclear antibody
anti-dsDNA: anti-double stranded DNA
EMG: Electromyography
CMAP: Compound muscle action potential
WES: Whole Exome Sequencing
PEO: Progressive external ophthalmoplegia
IBD: Inflammatory bowel disease
MRS: Magnetic resonance spectroscopy
HSCT: Hematopoietic stem cell transplantation
EE-TP: Erythrocyte encapsulated thymidine phosphorylase
